# Differentiation of volatile organic compounds in chili powders of different spiciness levels via E-nose, HS-GC–IMS, and chemometrics

**DOI:** 10.3389/fnut.2025.1629925

**Published:** 2025-07-28

**Authors:** Shibo Zhao, Meng Zhang, Yecheng Ran, Zhou Yang, Ruonan Dong, Linlin He, Wengang Jin, A. M. Abd El-Aty

**Affiliations:** ^1^Qinba State Key Laboratory of Biological Resources and Ecological Environment (Incubation), Shaanxi University of Technology, Hanzhong, China; ^2^School of Bioscience and Engineering, Shaanxi University of Technology, Hanzhong, China; ^3^Department of Pharmacology, Faculty of Veterinary Medicine, Cairo University, Giza, Egypt; ^4^Department of Medical Pharmacology, Medical Faculty, Ataturk University, Erzurum, Türkiye

**Keywords:** chili powder, electronic nose, headspace gas chromatography-ion mobility spectrometry, volatile organic compounds, chemometrics, relative odor activity values

## Abstract

**Introduction:**

Chili powder is a widely used seasoning whose pungency largely depends on its capsaicin content and volatile compounds.

**Methods:**

This study evaluated the capsaicin levels and pungency of three commercial chili powders labeled light, medium, and strong using a pungency meter. Volatile organic compounds (VOCs) were analyzed via electronic nose and headspace gas chromatography–ion mobility spectrometry (HS-GC–IMS) coupled with multivariate statistical analyses.

**Results:**

Capsaicin concentrations in the medium and strong chili powders were significantly greater than those in the light group (*p* < 0.01). The Scoville heat unit (SHU) values were 604 (light), 1,585 (medium), and 1733 (strong). The electronic nose successfully differentiated samples on the basis of spiciness level. HS-GC–IMS identified 48 VOCs, mainly aldehydes (51.74–55.55%) and ketones (29.93–32.09%). Variable importance projection (VIP > 1, *p* < 0.05) highlighted 21 marker volatiles, whereas fold change analysis (FC > 2 or < 0.5) identified 14 differential compounds across sample groups. Key odorants such as (E, E)-2,4-heptadienal, butanal, 3-methylbutanal, and 2,3-butanedione were associated with flavor differences among the chili powders.

**Conclusion:**

Chili powders with varying spiciness levels exhibit notable differences in capsaicin content, VOC profiles, and distinctive flavor markers, which can be effectively characterized through integrated sensory and chemical analyses.

## 1 Introduction

The chili pepper (*Capsicum annuum* L.), commonly known as hot pepper or bell pepper, belongs to the Solanaceae family and is recognized for its rich nutritional profile. It is particularly high in vitamin C—reportedly the highest among all vegetables—along with polyphenols, flavonoids, minerals, capsaicin, and carotenoids. These bioactive compounds have several health-promoting effects, including antioxidant, free radical-scavenging, and antitumor activities (1–3). In the spice industry, chili peppers are utilized in various forms, such as fresh chili, chili powder, chili sauce, and chili oil, which serve both functional and sensory purposes. Among these, chili powder is extensively used as a flavor enhancer in diverse culinary applications (2, 4). Recent studies have shown that chili powder not only delivers bioactive components such as capsaicin and carotenoids but also contributes distinctive colors, aromas, and tastes to food products (5, 6).

To date, numerous studies have focused on analyzing and characterizing the volatile flavor components of chili peppers. For example, Murakami et al. (6) utilized solvent extraction in combination with solvent-assisted flavor evaporation to isolate volatiles from Habanero peppers. Through GC–MS analysis, they identified 66 volatile compounds and attributed the characteristic aroma of Habanero chili to 6-methyl-(E)-4-heptenyl esters. Similarly, Mi et al. (7) applied GC–IMS to profile volatiles in different chili pepper genotypes and identified 18 differential volatile markers that distinguished Jize varieties from Korean chili varieties. These investigations highlight the use of volatile compound profiling to differentiate chili types on the basis of their aroma characteristics.

The electronic nose (E-nose), which uses a gas sensor array to mimic the human olfactory system, is widely employed in the food industry as a rapid and non-destructive detection tool. However, its ability to provide quantitative analyses is limited. In contrast, headspace gas chromatography–ion mobility spectrometry (HS-GC–IMS) has emerged as a powerful technique for identifying and characterizing volatile flavor compounds in food. Compared with solid-phase microextraction gas chromatography–mass spectrometry (SPME-GC–MS), HS-GC–IMS offers advantages in terms of simpler sample preparation. These two methods often detect differing sets and quantities of volatile compounds, with their sensitivity varying depending on the sample type (8, 9). Recent studies have combined HS-GC–IMS with SPME-GC–MS to achieve a more comprehensive analysis of food volatiles (10). Additionally, when paired with multivariate statistical approaches such as PLS-DA and OPLS-DA, HS-GC–IMS can effectively identify differential flavor markers among sample groups (11, 12). Its application in chili-based products has also been explored. For example, Hwang et al. (13) used HS-GC–IMS to compare VOC profiles between freshly prepared and stored chili powders, constructing fingerprint maps and identifying key differential volatiles. While both the E-nose and HS-GC–IMS provide valuable insights into aroma profiles, each contributes uniquely to understanding the volatile composition of food samples (14, 15).

The sensory flavor of chili powder is strongly influenced by its capsaicin content and degree of spiciness (13, 16). Capsaicin, the primary pungent compound in chili peppers, stimulates sensory nerves in the mouth and skin to evoke a burning sensation (17), while it also plays a significant role in shaping the volatile flavor profile of chili powder. Despite its importance, few studies have investigated the differences in aroma components among chili powders with varying spiciness levels.

On the basis of the above considerations, we hypothesized that specific volatile organic compounds (VOCs) differ significantly among chili powders with varying levels of spiciness, thereby influencing their sensory characteristics. Accordingly, this study aimed to assess the capsaicin content and pungency levels of commercially available chili powders labeled as light, medium, or strong. Additionally, differences in VOC profiles were characterized via an electronic nose (E-nose) and headspace gas chromatography–ion mobility spectrometry (HS-GC–IMS). These findings provide a theoretical basis for the quality control and flavor profiling of retail chili powder products.

## 2 Materials and methods

### 2.1 Materials

Three commercially available chili powders with distinct spiciness levels—light, medium, and strong—were freshly prepared from dehydrated Qinjiao pepper varieties harvested in September 2024. The powders were sourced from the Lianhu Farmers’ Market in Hanzhong, China, in mid-December 2024. On the basis of product labels and vendor descriptions, the chili powders were classified and stored at 4°C according to their pungency levels: light spicy as Weila (WL), medium spicy as Zhongla (ZL), and strong spicy as Tela (TL), corresponding to their Chinese designations.

C4-C9 n-ketones (2-butanone, 2-pentanone, 2-hexanone, 2-heptanone, 2-octanone, and 2-nonanone) were all analytically pure and obtained from National Pharmaceutical Group Chemical Reagent Co., Ltd., Beijing.

### 2.2 Capsaicin content and spiciness level determination

The capsaicin content and Scoville heat unit (SHU) values were measured via an HM-LD pungency meter (Shandong Hengmei Electronic Technology Co., Ltd., China). A total of 0.5 g of ground chili powder was weighed, followed by the addition of 5 mL of anhydrous ethanol. The mixture was vortexed for 5 min to ensure thorough extraction. Subsequently, 1 mL of the extract was centrifuged via a MySPIN™ 12 centrifuge (Thermo Fisher Scientific Inc., Waltham, United States), and 100 μL of the resulting supernatant was collected. To this mixture, 400 μL of the Pungency Test Kit reagent (Shandong Hengmei) was added, and the mixture was mixed thoroughly. Then, 50 μL of the final mixture was applied to the electrode strip of the HM-LD meter (Hengmei Electronic Technology Co., Ltd., China) for triplicate measurements. The device automatically reported the capsaicin concentration and corresponding SHU value.

### 2.3 E-nose analysis

E-nose analysis was performed according to the slightly modified method of Sun et al. (18). A 2 g sample was accurately weighed into a 20 mL vial, which was sealed with a cap and equilibrated at 25°C for 30 min. The samples were subsequently tested via a PEN3 E-nose instrument. The sensors are sensitive to different compounds, including aromatic compounds (W1C), nitrogen oxides (W5S), ammonia and aromatic compounds (W3C), hydrogen compounds (W6S), alkanes and aromatic compounds (W5C), methane (W1S), sulfur compounds (W1W), alcohols (W2S), aromatics and organic sulfur compounds (W2W), and long-chain alkanes (W3S). The instrument self-cleaning time was 100 s, the detection time was 200 s, and the data were collected from 196 to 198 s. The samples were prepared in triplicate and analyzed separately.

### 2.4 HS-GC–IMS detection of volatile organic compounds

The VOCs in the chili powder samples were analyzed via an HS-GC–IMS FlavorSpec^®^ flavor analyzer (G. A. S. Company, Germany) following the procedures described in our previous methods (19). A 3.0 g sample of each chili powder sample was accurately weighed and placed in a 20.0 mL headspace sampling vial. The samples were incubated at 80°C for 5 min, and then 500 μL of the headspace was injected into a FlavorSpec^®^ flavor analyzer (G. A. S. Company, Germany) to analyze the volatile organic compounds in the samples. The six n-ketones mentioned earlier were used as external standards to determine the relative content of volatile compounds, utilizing the retention time (RT), retention index (RI), and drift time (DT) data from the instrument’s library. The ROAV was calculated via the following equation:

$$\text{ROAV}(\%)=\frac{C_{i}}{C_{s}}\times \frac{T_{s}}{T_{i}}\times 100$$

where *C_i_* and *T_i_* represent the relative content and threshold value of each volatile substance in the chili powder samples, respectively; *C_s_* represents the relative content of the volatile substance that contributes the most to the flavor of the sample; and *T_s_* represents its threshold value (20).

### 2.5 Data processing

Each sample was tested in parallel three times. 3D and 2D spectra and fingerprint maps were exported via LAV, Reporter, Gallery Plot, and other plugins of the flavor analyzer. The identification of aroma compounds was performed via the NIST2020 and IMS databases. The relative amounts of different volatile components were determined via normalized peak volumes. Significance analysis was performed via SPSS (*p* < 0.05). R Studio (psych, ggplot2 package) and the online websites^1^,^2^ were used for multicomponent volcano plots, principal component analysis (PCA), box plots, radar of sensory aroma, and heatmap creation (21).

## 3 Results and discussion

### 3.1 Capsaicin content and spiciness level

**Figure 1:**
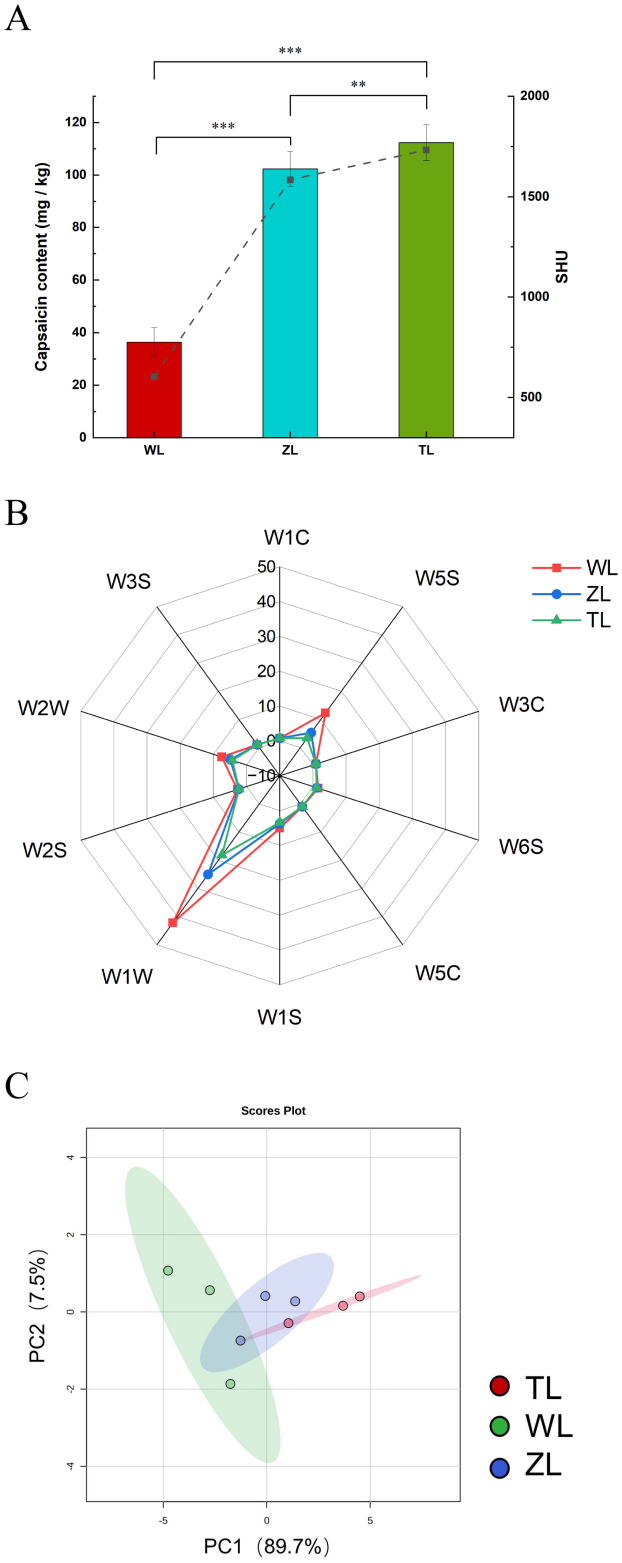
Chili powders with different spiciness levels. **(A)** Capsaicin content and spiciness level (Significance between groups: **p < 0.01, ***p < 0.001). **(B)** Electronic nose radar chart. The sensors are sensitive to different compounds, including aromatic compounds (W1C), nitrogen oxides (W5S), ammonia and aromatic compounds (W3C), hydrogen compounds (W6S), alkanes and aromatic compounds (W5C), methane (W1S), sulfur compounds (W1W), alcohols (W2S), aromatics and organic sulfur compounds (W2W), and long-chain alkanes (W3S). **(C)** Electronic nose PCA.

Figure 1A presents the capsaicin content of the three types of chili powders. Compared with the WL samples, both the ZL and TL samples presented significantly higher capsaicin levels, with notable differences observed between the groups (*p* < 0.01). The Scoville heat unit (SHU) value is widely used to represent the pungency or spiciness of chili peppers. The SHU values for the WL, ZL, and TL groups were 604, 1,585, and 1733, respectively (Figure 1A), which were consistent with the labeled spicy levels of the chili powders used. The differences in capsaicin content among the groups resulted in variations in SHU values, with the TL group exhibiting the highest value. Capsaicin, the key bioactive agent responsible for spicy sensation in chili peppers, is influenced primarily by a combination of genetic and environmental factors. Its concentration can vary significantly depending on the chili cultivar., cultivation conditions, postharvest storage, and developmental stage of the fruit. For example, a comparative study of four varieties—‘Habanero’, ‘Habanero Roxo’, ‘Bode’, and ‘Malagueta’—demonstrated that capsaicin content peaks during a specific growth phase and subsequently decreases. These dynamic changes are closely tied to the fruit’s genotype and external growth conditions, highlighting the complexity of capsaicinoid biosynthesis and accumulation (22). Capsaicin also has pharmacological effects, such as analgesic, anticancer, anti-inflammatory, and antiobesity effects (23). Additionally, dihydrocapsaicin, similar to capsaicin, is pungent and contributes to the spiciness of chili peppers (23, 24).

Studies have shown that capsaicin has certain antibacterial properties that may affect the final flavor of peppers. For example, Zhang et al. (25) reported that under capsaicin stress, the varying activities of lactic acid bacteria can lead to changes in the quality and flavor of fermented pepper products. This research hypothesizes that capsaicin stress can promote *Lactobacillus plantarum* production of substances such as acids and esters. Shi et al. (26) reported that in chili sauce, capsaicin and fermentation time resulted in variations in Bacteroides and Kazachstania. Additionally, capsaicin stress significantly decreased microbial diversity, richness, and the number of observed species, and nonanoate esters are known as key substances that respond to capsaicin stress. However, a study by Ziino et al. (27) revealed that there is no clear correlation between the content of VOCs and pungency in pepper samples purchased from the Calabrian Producers Association.

### 3.2 E-nose analysis

The overall flavor of the chili powders was determined via an electronic nose, and the sensor response values of three chili powders with different spiciness levels were plotted in a radar chart, as shown in Figure 1B. The 10 axes represent 10 sensors. The chili powders presented the greatest response to W1W, followed by W5S and W2W. The radar chart areas of the three chili powders varied. WL strongly responded to W1W, followed by ZL and finally TL. W1W primarily detects terpenes and sulfur-containing organic compounds, indicating that WL contains relatively high amounts of corresponding compounds. WL also strongly responded to W5S and W2W, which are sensitive to nitrogen oxides, aromatic compounds, and organic sulfur compounds, although the changes were small. The responses of the other sensors showed minimal changes, indicating weak volatility of the corresponding components. Sun et al. (18) also detected differences between groups of braized chicken samples at different stages of stewing, which resulted in distinct radar charts. To further observe the odor variations of chili powders with different spiciness levels, PCA was performed, as shown in Figure 1C. PCA is an unsupervised pattern recognition method for multivariate data analysis, which corresponds to the first and second principal components, respectively (28). PC1 and PC2 explained 97.2% of the variance, indicating that they can effectively summarize and reflect the variations in the aroma of the chili powder. The factors contributing to the differences in chili flavor between groups include variations in the content of capsaicinoids or volatile compounds (23).

### 3.3 HS-GC–IMS spectra of the volatile components of the three types of chili powder

**Figure 2:**
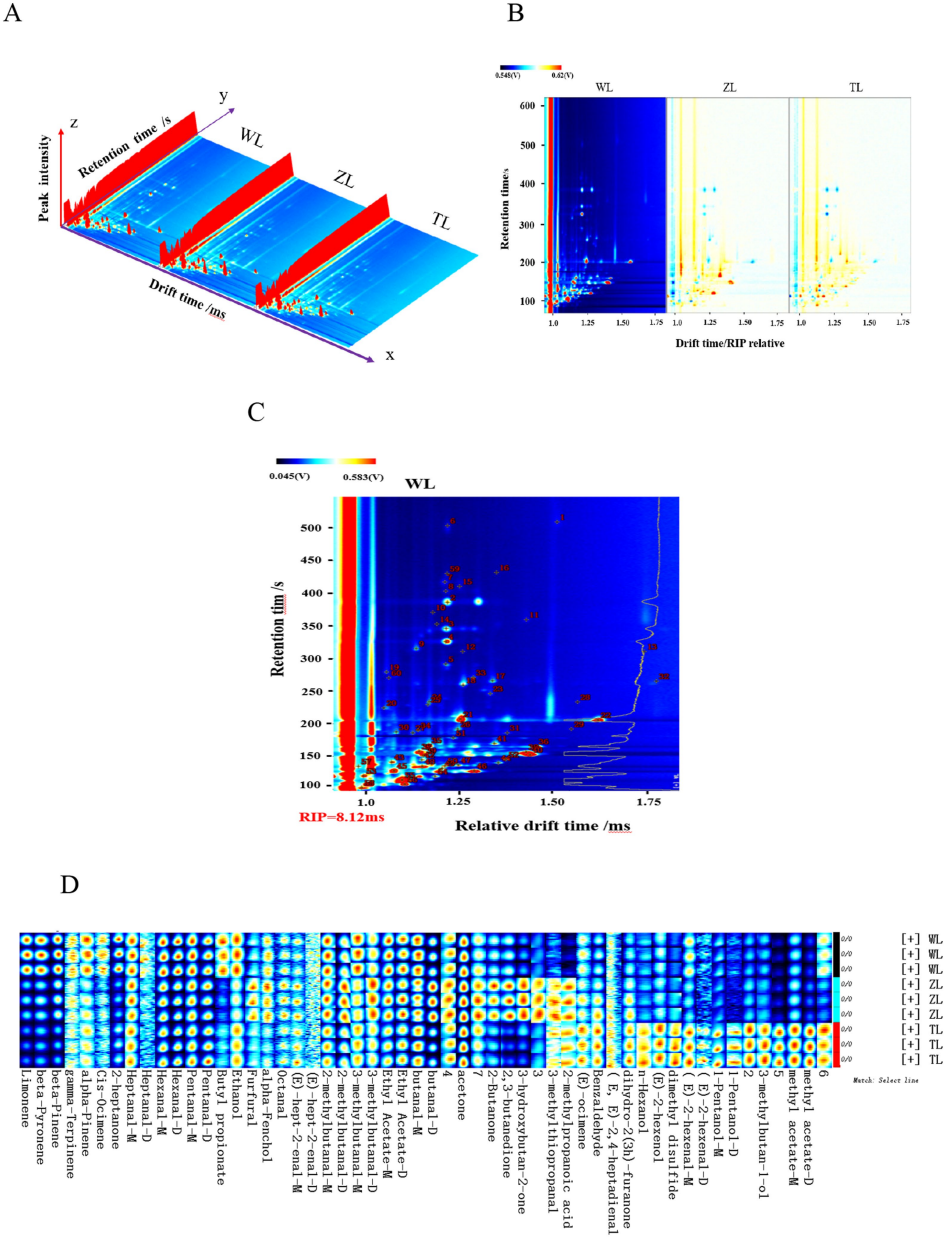
**(A)** 3D map of the HS-GC–IMS spectra. **(B)** 2D comparison map. **(C)** Qualitative spectra of volatile components in lightly spicy (WL) chili powder. **(D)** Fingerprints of volatile organic compounds. The lightly spicy chili powder was labeled Weila (WL), the medium spicy chili powder was labeled Zhongla (ZL), and the strongly spicy chili powder was labeled Tela (TL) in Chinese.

HS-GC–IMS was employed to analyze the VOCs in three types of chili powder with different spiciness levels (slight, medium, and strong). Figure 2A shows three-dimensional topographic maps of volatile components in chili powder samples with different spiciness levels, with each point representing a VOC and the color indicating the signal intensity (13). The three-dimensional maps, from left to right, correspond to slight, medium, and strong (denoted by WL, ZL, and TL, respectively, in Chinese), chili powders. The differences in volatile substances in the three-dimensional maps of chili powders with different spiciness levels were not easily comparable by the naked eye and require further dimensionality reduction to better present the subtle differences in VOCs in chili powders with different spiciness levels.

Figure 2B shows a two-dimensional differential comparison plane obtained by projecting three-dimensional topographic maps of chili powders with different spiciness levels (with slight chili powder used as a control to subtract the background), which makes it relatively easy to compare the subtle differences in volatile components among chili powders with different spiciness levels. GC–IMS efficiently separated the VOCs in the chili powder samples, and the contents of some of the VOCs in the chili powders varied, indicating certain differences. Consequently, the HS-GC–IMS characteristic spectra of the chili powders also exhibited partial differences, and further qualitative and quantitative analyses were subsequently conducted.

### 3.4 Qualitative analysis of volatile organic compounds in chili powders with different spiciness levels via HS-GC–IMS

In HS-GC–IMS analyses of qualitative flavor components, ketones C4-C9 are typically employed as external standards. By analyzing the retention and migration times of different volatile components, the retention indices of the VOCs were obtained. These indices are then matched against the instrument database to conduct qualitative analyses of volatile aromas (21). Figure 2C shows the qualitative spectra of the VOCs in the WL chili powders. By utilizing the NIST 2014 database integrated into the GC–IMS instrument, along with the G. A. S. IMS migration time database, 48 VOCs were identified from the 60 signal peaks in the three chili powder samples with different spiciness levels, including 21 aldehydes, 7 alcohols, 7 terpenes, 6 esters, 5 ketones, 1 ether, and 1 acid. The retention indices (RIs), retention times (RTs), relative contents, and odor characteristics of various identified VOCs are displayed in Table 1. The acetone content was the highest among the three chili powder samples, followed by 2-methylbutanal-D, butanal, 3-methylbutanal-D, and hexanal. The relative contents of other VOCs also differed among the three chili powder samples with different spiciness levels. For example, the contents of limonene, β-pyronene, β-pinene, and butyl propionate tended to decrease, whereas the contents of other substances, such as n-hexanol, dimethyl disulfide, 3-methyl-1-butanol, and methyl acetate dimers, increased (Table 1).

**Table 1:** Volatile organic compounds identified in chili powder with different levels of spiciness.

No	Compounds	CAS	RI	RT/s	DT/ms	Relative amount/%		
						WL	ZL	TL
1	limonene	C138863	1024.2	385.77	1.22436	2.33 ± 0.02^a^	1.07 ± 0.06^b^	0.90 ± 0.06^c^
2	β-pyronene	C514965	996.1	345.597	1.22169	1.51 ± 0.02^a^	0.84 ± 0.04^b^	0.73 ± 0.02^c^
3	β-pinene	C127913	973	325.886	1.2208	3.10 ± 0.04^a^	2.10 ± 0.06^b^	1.44 ± 0.02^c^
4	α-pinene	C80568	932.8	291.604	1.21996	0.28 ± 0.00^a^	0.18 ± 0.01^b^	0.20 ± 0.02^b^
5	α-fenchol	C512130	1105.7	502.93	1.22455	0.36 ± 0.01^a^	0.33 ± 0.02^a^	0.29 ± 0.00^b^
6	(*E*)-ocimene	C3779611	1045.9	417.013	1.21835	0.30 ± 0.03a^b^	0.25 ± 0.02^b^	0.34 ± 0.03^a^
7	cis-ocimene	C3338554	1036.2	403.064	1.22077	0.16 ± 0.01^a^	0.11 ± 0.01^b^	0.11 ± 0.01^b^
8	Benzaldehyde	C100527	960.2	314.942	1.15319	0.74 ± 0.02^c^	1.00 ± 0.15^b^	1.47 ± 0.05^a^
9	(*E*, E)-2,4-heptadienal	C4313035	1013.7	370.628	1.19115	0.35 ± 0.00^a^	0.33 ± 0.03^a^	0.34 ± 0.02^a^
10	Octanal	C124130	1005.7	359.213	1.40373	0.29 ± 0.01^b^	0.29 ± 0.02^b^	0.33 ± 0.01^a^
11	(*E*)-hept-2-enal-M	C18829555	955.1	310.623	1.25948	0.25 ± 0.04^a^	0.23 ± 0.01^a^	0.29 ± 0.05^a^
12	(*E*)-hept-2-enal-D	C18829555	954.9	310.469	1.67271	0.09 ± 0.00^b^	0.09 ± 0.01^b^	0.12 ± 0.01^a^
13	Heptanal-M	C111717	902.2	265.469	1.32689	0.57 ± 0.01^a^	0.47 ± 0.01^b^	0.59 ± 0.01^a^
14	2-heptanone	C110430	894.7	259.097	1.26038	1.47 ± 0.01^a^	0.77 ± 0.05^c^	1.14 ± 0.02^b^
15	Dihydro-2(3H)-furanone	C96480	918.2	279.093	1.08527	0.22 ± 0.01^b^	0.24 ± 0.02^b^	0.36 ± 0.01^a^
16	Furfural	C98011	831.5	224.378	1.07984	0.34 ± 0.02^a^	0.35 ± 0.02^a^	0.33 ± 0.01^a^
17	Hexanal-M	C66251	799	206.983	1.25384	7.49 ± 0.05^a^	5.52 ± 0.10^c^	6.45 ± 0.06^b^
18	Hexanal-D	C66251	797.1	205.965	1.56695	5.58 ± 0.02^a^	3.75 ± 0.15^c^	4.80 ± 0.06^b^
19	n-hexanol	C111273	871.9	246.032	1.32154	0.25 ± 0.02^b^	0.18 ± 0.01^c^	0.42 ± 0.04^a^
20	(*E*)-2-hexenal-M	C6728263	849.3	233.953	1.18297	0.25 ± 0.01^b^	0.18 ± 0.00^c^	0.33 ± 0.02^a^
21	(*E*)-2-hexenol	C928950	840.8	229.414	1.18024	0.12 ± 0.00^b^	0.08 ± 0.00^c^	0.18 ± 0.01^a^
22	1-pentanol-M	C71410	766.5	191.874	1.24766	0.96 ± 0.03^b^	0.65 ± 0.03^c^	1.58 ± 0.05^a^
23	Dimethyl disulfide	C624920	752.5	186.191	1.1454	0.45 ± 0.00^c^	0.56 ± 0.04^b^	1.17 ± 0.05^a^
24	(*E*)-2-hexenal-D	C6728263	847.9	233.202	1.52111	0.04 ± 0.00^b^	0.04 ± 0.00^b^	0.06 ± 0.00^a^
25	1-pentanol-D	C71410	765.6	191.529	1.50672	0.07 ± 0.00^b^	0.06 ± 0.01^b^	0.17 ± 0.01^a^
26	Heptanal-D	C111717	901.9	265.22	1.70043	0.06 ± 0.01^a^	0.06 ± 0.01^a^	0.06 ± 0.01^a^
27	Butyl propionate	C590012	908.5	270.821	1.28338	0.14 ± 0.00^a^	0.06 ± 0.01^c^	0.09 ± 0.00^b^
28	2-methylpropanoic acid	C79312	761.8	189.991	1.15788	0.06 ± 0.00^c^	0.16 ± 0.01^b^	0.19 ± 0.01^a^
29	Pentanal-M	C110623	705.1	166.977	1.18187	2.90 ± 0.01^a^	2.26 ± 0.05^c^	2.63 ± 0.06^b^
30	Pentanal-D	C110623	701.6	165.558	1.42608	2.18 ± 0.03^ab^	1.97 ± 0.08^b^	2.36 ± 0.20^a^
31	2-methylbutanal-M	C96173	678.4	157.549	1.15929	3.26 ± 0.01^a^	2.65 ± 0.04^c^	2.86 ± 0.09^b^
32	2-methylbutanal-D	C96173	675.4	156.738	1.40279	8.03 ± 0.13^b^	10.92 ± 0.16^a^	8.07 ± 0.03^b^
33	3-methylbutanal-M	C590863	656.6	151.669	1.16988	2.62 ± 0.04^a^	2.05 ± 0.07^b^	2.59 ± 0.03^a^
34	3-methylbutanal-D	C590863	659.6	152.48	1.41196	6.29 ± 0.07^c^	7.73 ± 0.08^a^	6.93 ± 0.03^b^
35	3-hydroxybutan-2-one	C513860	711.6	169.613	1.3315	0.64 ± 0.02^b^	2.09 ± 0.07^a^	0.75 ± 0.15^b^
36	Ethyl acetate-M	C141786	618.4	141.348	1.09716	1.27 ± 0.04^a^	1.18 ± 0.03^b^	1.23 ± 0.02a^b^
37	Ethyl acetate-D	C141786	614.7	140.361	1.34015	0.56 ± 0.02^a^	0.54 ± 0.04^a^	0.58 ± 0.03^a^
38	Butanal-M	C123728	569.1	128.066	1.10289	4.32 ± 0.02^a^	3.87 ± 0.03^c^	4.20 ± 0.05^b^
39	Butanal-D	C123728	567.5	127.618	1.28571	7.29 ± 0.11^b^	11.76 ± 0.2^a^	6.91 ± 0.05^c^
40	2-butanone	C78933	600.7	136.592	1.24903	0.53 ± 0.00^b^	0.73 ± 0.03^a^	0.53 ± 0.01^b^
41	2,3-butanedione	C431038	592.8	134.438	1.16708	2.33 ± 0.04^b^	3.25 ± 0.10^a^	1.39 ± 0.06^c^
42	3-methylbutan-1-ol	C123513	732.8	178.195	1.23769	0.22 ± 0.00^b^	0.22 ± 0.01^b^	0.49 ± 0.02^a^
43	Methyl acetate-M	C79209	539.4	120.056	1.03263	1.18 ± 0.01^b^	1.04 ± 0.03^c^	2.20 ± 0.03^a^
44	Methyl acetate-D	C79209	537.8	119.634	1.1963	0.57 ± 0.01^b^	0.63 ± 0.04^b^	2.54 ± 0.03^a^
45	Acetone	C67641	516	113.743	1.12092	24.97 ± 0.20^b^	25.25 ± 0.16^b^	26.61 ± 0.03^a^
46	Ethanol	C64175	492.4	107.378	1.12709	2.79 ± 0.01^a^	1.76 ± 0.07^c^	2.47 ± 0.03^b^
47	*γ* –terpinene	C99854	1054.7	429.647	1.22305	0.18 ± 0.02^a^	0.14 ± 0.01^ab^	0.14 ± 0.02^b^
48	3-methylthiopropanal	C3268493	907.5	269.972	1.09011	0.02 ± 0.00^a^	0.03 ± 0.01^a^	0.03 ± 0.00^a^

The suffix D in the table represents dimers of volatile organic compounds, whereas M represents monomers. Different lowercase letters with superscripts in the same row denote significant differences between samples (*p* < 0.05). The odor descriptions are from www.femaflavor.org and www.goodscentscompany.com.

### 3.5 Fingerprint characteristics of volatile organic compounds in chili powders with different spiciness levels

Three parallel measurements were performed on the three chili powder samples with different spiciness levels to obtain the HS-GC–IMS spectra of all the VOCs. The fingerprints of VOCs in chili powders with different spiciness levels were visualized and constructed via the built-in plugins of the instrument (Figure 2D). The horizontal axis shows the different spiciness levels of chili powders (from top to bottom, slightly spicy, moderately spicy, and strongly spicy are denoted by WL, ZL, and TL, respectively, in Chinese), whereas the vertical axis shows the specific identified volatile organic compounds in the chili powders (29).

Comparisons along the horizontal and vertical axes in Figure 2D revealed significant differences in flavor components among the three chili powders with different spiciness levels. For example, slightly specified chili powder (WL) has relatively high contents of limonene, β-pyronene, β-pinene, 2-heptanone, butyl propionate, and ethanol. The medium chili powder (ZL) had relatively high contents of furfural, 2-methylpropionic acid, 3-methylbutanal-D, 3-hydroxybutan-2-one, 2-butanone, 2,3-butanedione, and 3-methylthiopropanal. The strong spiciness chili powder (TL) had relatively high contents of (E)-ocimene, benzaldehyde, n-hexanol, dimethyl disulfide, 1-pentanol, 3-methylbutan-1-ol, and methyl acetate. The differences in the characteristic VOCs of these three types of chili powders with different spiciness levels might influence the overall volatile flavor characteristics of each chili powder.

**Figure 3:**
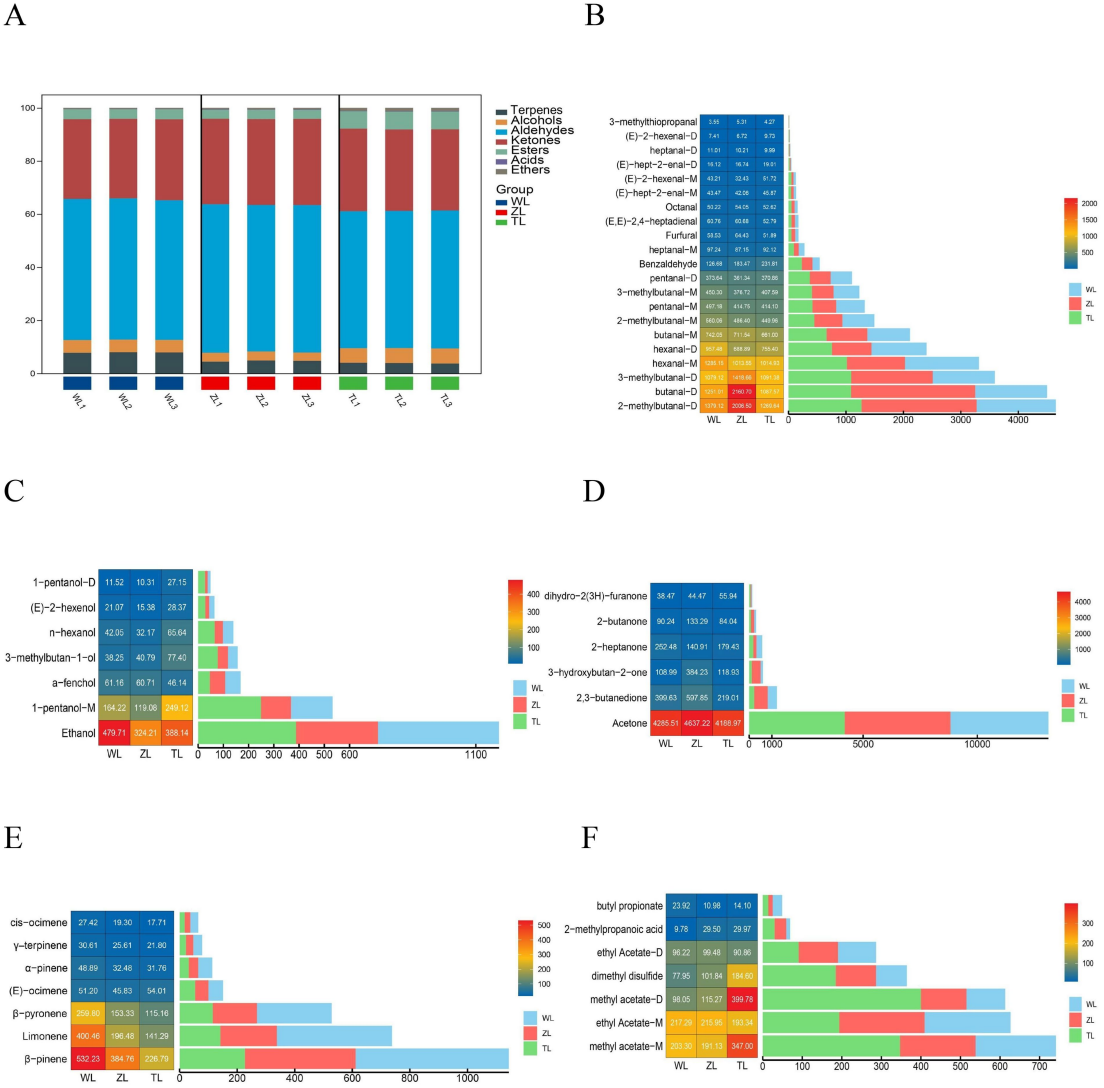
**(A)** Relative contents of various volatile organic compounds in chili powders with different spicy contents. **(B)** Peak volumes of aldehydes. **(C)** Peak volumes of alcohols. **(D)** Peak volumes of ketones. **(E)** Peak volumes of terpenes. **(F)** Peak volumes of esters, acids and ethers. The lightly spicy chili powder was labeled Weila (WL), the medium spicy chili powder was labeled Zhongla (ZL), and the strongly spicy chili powder was labeled Tela (TL) in Chinese.

During the thermal processing of food ingredients, a variety of complex volatile flavor components, such as aldehydes, alcohols, ketones, esters, furans, and ethers, are produced (30–33). To clearly present the overall profile of various volatile flavor substances in the three types of chili powders, the relative proportions of various volatile flavor substances were calculated on the basis of the signal intensity of each organic compound in the fingerprint spectrum, normalized by peak volume. Figure 3A shows that the volatile flavor substances at the three spiciness levels included aldehydes, ketones, alcohols, esters, terpenes, acids, and ethers, with aldehydes and ketones being the most predominant.

Figures 3B–F categorizes all the compounds and displays detailed peak volumes for each category. The concentrations of detected aldehydes in the WL, ZL, and TL groups are shown, with the *x*-axis representing peak volume and the *y*-axis listing compound names. Additionally, a heatmap on the left indicates relative compound amounts, with numbers representing average peak volumes of replicates. 2-Methylbutanal-D and butanal-D are among the aldehydes present at relatively high concentrations (Figure 3B). Among the alcohols, ethanol had the highest concentration (Figure 3C), acetone was the most abundant (Figure 3D), and among the terpenes, β-pinene had the highest concentration (Figure 3E). Finally, Figure 3F shows all esters, acids, and ethers, with methyl acetate-M being the most abundant in the ester category. A study on chili oil indicated that its primary volatile compounds are aldehydes, alcohols, and ketones, with (*E, E*)-2,4-heptadienal being the volatile aldehyde with the highest relative content, and we also detected this compound in this analysis (29). While previous studies on chili oil have identified a range of aldehydes, our findings additionally revealed the presence of several other aldehydes, including 2-methylbutanal-D and butanal. Moreover, the overall proportions of ketones and alcohols identified in our samples differed from those typically reported in chili oil. These differences are likely attributed to several factors. First, variations in processing methods play a key role—specifically, the heating process can induce thermal oxidative degradation, where saturated fatty acids undergo oxidation to form alkenes and alcohols, which may further oxidize at elevated temperatures to yield ketones (5, 34). Second, differences in pepper species can influence the compound profile; for example, Trovato et al. (35) reported distinct volatile compounds across three pepper varieties: *Capsicum chinense*, *C. annuum*, and *C. baccatum*. Finally, discrepancies in analytical instrumentation and detection methodologies may also account for variations in VOC identification across studies (36). Taken together, these findings suggest that variations in volatile compound profiles arise from a combination of processing techniques, pepper varieties, and analytical approaches.

### 3.6 Multivariate statistical analysis of chili powders with different spiciness levels

**Figure 4:**
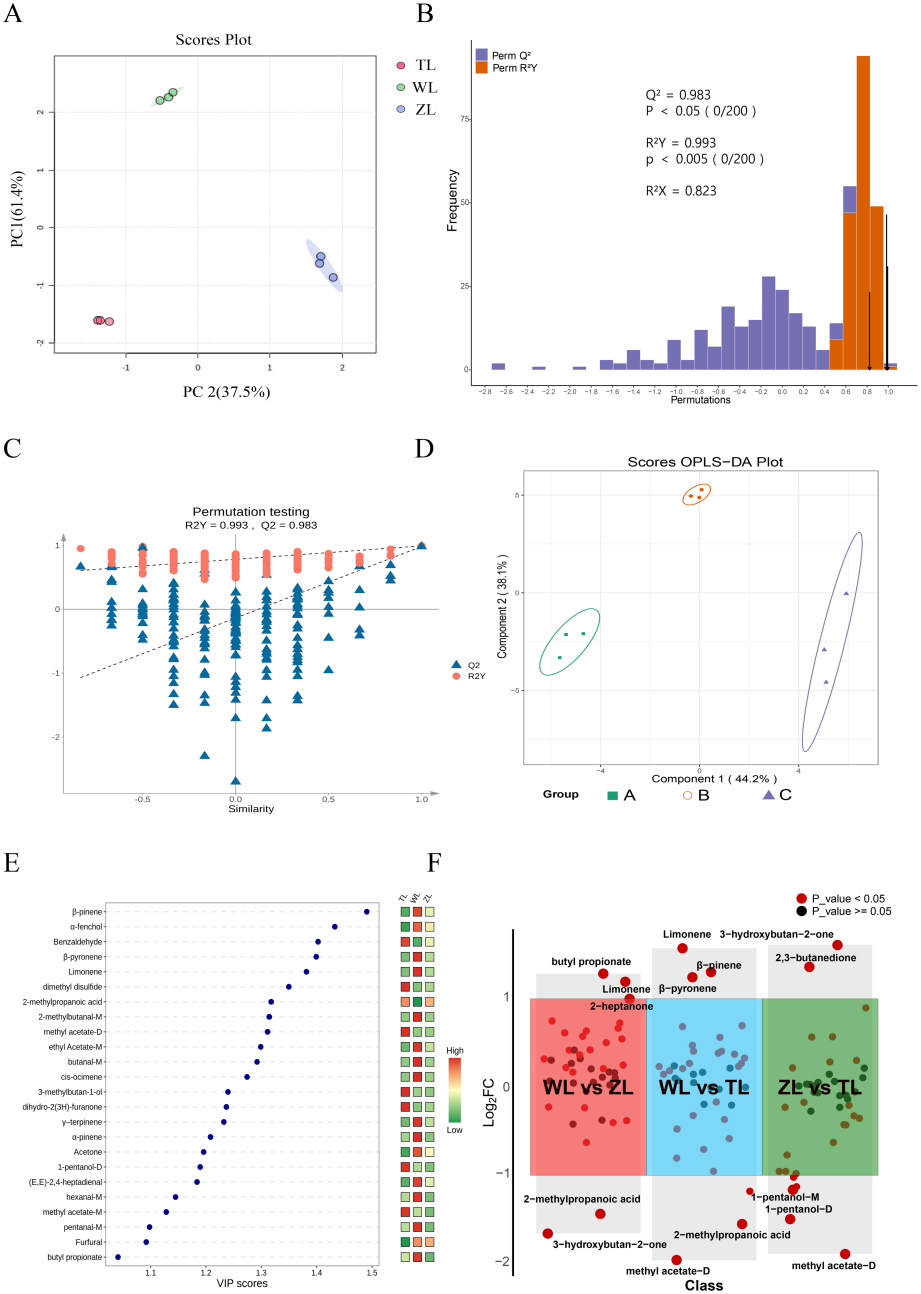
Multivariate statistical analysis of chili powders at different spiciness levels: **(A)** PCA scores. **(B)** OPLS-DA model validation diagram. **(C)** Permutation analysis plotting *R*^2^ and *Q*^2^ from two hundred permutation tests in the OPLS-DA model. **(D)** Two-dimensional OPLS-DA plot. **(E)** VIP score. **(F)** Volcano plot (WL vs. ZL, WL vs. TL, ZL vs. TL; all compounds included in the comparison had VIP > 1, *p* < 0.05, and fold change (FC) > 2 or < 0.5).

Capsaicin determines the spiciness of chili peppers, whereas the types and concentrations of aroma compounds determine the aroma and flavor complexity of chili powders. To determine the compositional differences in aroma compounds among chili powder samples of varying spiciness, PCA was conducted (Figure 4A). Each principal component’s corresponding percentage indicates how much of the total variance in the data it explains. Figure 4A shows that PC1 and PC2 together explained 98.9% of the variance, demonstrating that these principal components effectively capture the major differences among the flavor compounds in the chili powders.

Furthermore, OPLS-DA was employed. It is a statistical method designed for pattern recognition and data classification and is particularly effective with high-dimensional data. It is a variant of PLS-DA that incorporates orthogonal signal correction to enhance the model’s predictive capabilities and interpretability (37). In OPLS-DA, *R^2^X* and *R^2^Y* denote the proportion of variance explained by the model in the X and Y matrices, respectively, whereas *Q^2^* reflects its predictive performance. Values of these metrics approaching 1 suggest a stable and reliable model. Typically, a *Q^2^* exceeding 0.5 indicates a valid model, and a *Q^2^* above 0.9 signifies an outstanding model. As shown in Figure 4B, *R^2^X* = 0.823, *R^2^Y* = 0.993, and *Q^2^* = 0.983, indicating that the model has good robustness and predictive ability (38).

The reliability of OPLS-DA was assessed through 200 permutation tests on the model (Figure 4C) to verify that the model was not overfit. This method assesses whether the original model possesses true discriminative power by repeatedly randomizing the class labels of the samples (39). The orange circles represent the *R^2^Y* values for each permutation, and the blue triangles represent the *Q^2^* values. The vertical axis shows the values of *R^2^Y* and *Q^2^*, whereas the horizontal axis represents the degree of permutation retention (0 for random permutation, 1 for the original value). The dashed lines denote the corresponding fitted regression lines for the observed and permutated *R^2^* and *Q^2^* values (40). In most cases of permutation, the *R^2^Y* and *Q^2^* values are lower than the original values (at a permutation retention degree of 1), indicating that the model is highly robust (Figure 4C). Both our study and that of Meng et al. (41) implemented OPLS-DA for multivariate statistical analysis in different contexts. Specifically, they used OPLS-DA to analyze the VOCs of *Dictyophora rubrovalvata* under various drying temperatures and reported that 80°C was the ideal temperature. Similarly, we apply this analytical method to assess the reliability of our model, demonstrating its versatility across diverse research settings.

Using a two-dimensional OPLS-DA plot, we observed separation between groups of chili powders with different levels of spiciness (Figure 4D). Additionally, VIP scores were used to identify the most influential variables for distinguishing between different levels of spiciness, providing a measure of the importance of each variable within the model. Generally, VIPs > 1 are considered particularly important to the model, with higher VIP values indicating greater importance (42). As illustrated in Figure 4E, each point represents a compound, with its position (horizontally) showing the VIP score. The color bar on the right side of the graph (ranging from green to red) represents the relative peak volume of each compound, where red indicates high and green indicates low. The graph displays all 24 flavor compounds with VIP > 1, including β-pinene, α-fenchol benzaldehyde, β-pyronene, limonene, dimethyl disulfide, 2-methylpropanoic acid, 2-methylbutanal-M, methyl acetate-D, ethyl acetate-M, butanal-M, cis-ocimene, 3-methylbutan-1-ol, dihydro-2(3H)-furanone, γ-terpinene, α-pinene, acetone, 1-pentanol-D, (*E, E*)-2,4-heptadienal, hexanal-M, methyl acetate-M, pentanal-M, furfural and butyl propionate (Figure 4E). These volatile substances include terpenes such as β-pinene, α-pinene, and γ-terpinene, which are found in the essential oils of many plants, such as *Ocimum menthaefolium*, *Pinus* spp., and *Juniperus communis*, and have a wide range of medicinal value (43, 44). Most esters (ethyl acetate and butyl propionate) typically provide fruity, floral, and sweet notes (45). Additionally, it contains aldehydes with low thresholds, which significantly contribute to flavor.

Additionally, by using FC > 2 or < 0.5 (*p* < 0.05), the chili powders of three different spiciness levels were pairwise compared (WL vs. ZL, WL vs. TL, ZL vs. TL) to observe substances that were upregulated or downregulated. The volcano plot (Figure 4F) displays the comparison groups on the horizontal axis and the log_2_FC values of the differentially expressed substances in each comparison group on the vertical axis. Substances are considered non-significant when the compound’s FC values are between 0.5 and 2 (log_2_FC > −1 and log_2_FC < 1). In the first comparison (WL vs. ZL), the substances that were upregulated (log_2_FC > 1) included limonene, 2-heptanone, and butyl propionate; those that were downregulated (log_2_FC < −1) included 2-methylpropanoic acid and 3-hydroxybutan-2-one. In the second comparison (WL vs. TL), substances that were upregulated included limonene, β-pyronene, and β-pinene; those that were downregulated included dimethyl disulfide, 1-pentanol-D, 2-methylpropanoic acid, and methyl acetate-D. In the third comparison (ZL vs. TL), substances that were upregulated included 3-hydroxybutan-2-one and 2,3-butanedione; those that were downregulated included *n*-hexanol, 1-pentanol-M, 1-pentanol-D, 3-methylbutan-1-ol, and methyl acetate-D. Volcano plots are commonly used to display the results of pairwise comparisons and are widely utilized in gene expression data analysis (46) and omics studies (47). Moreover, the use of set fold changes and VIP thresholds allows for the selection of differential substances between different groups, providing reliable data for further analysis (48).

**Figure 5:**
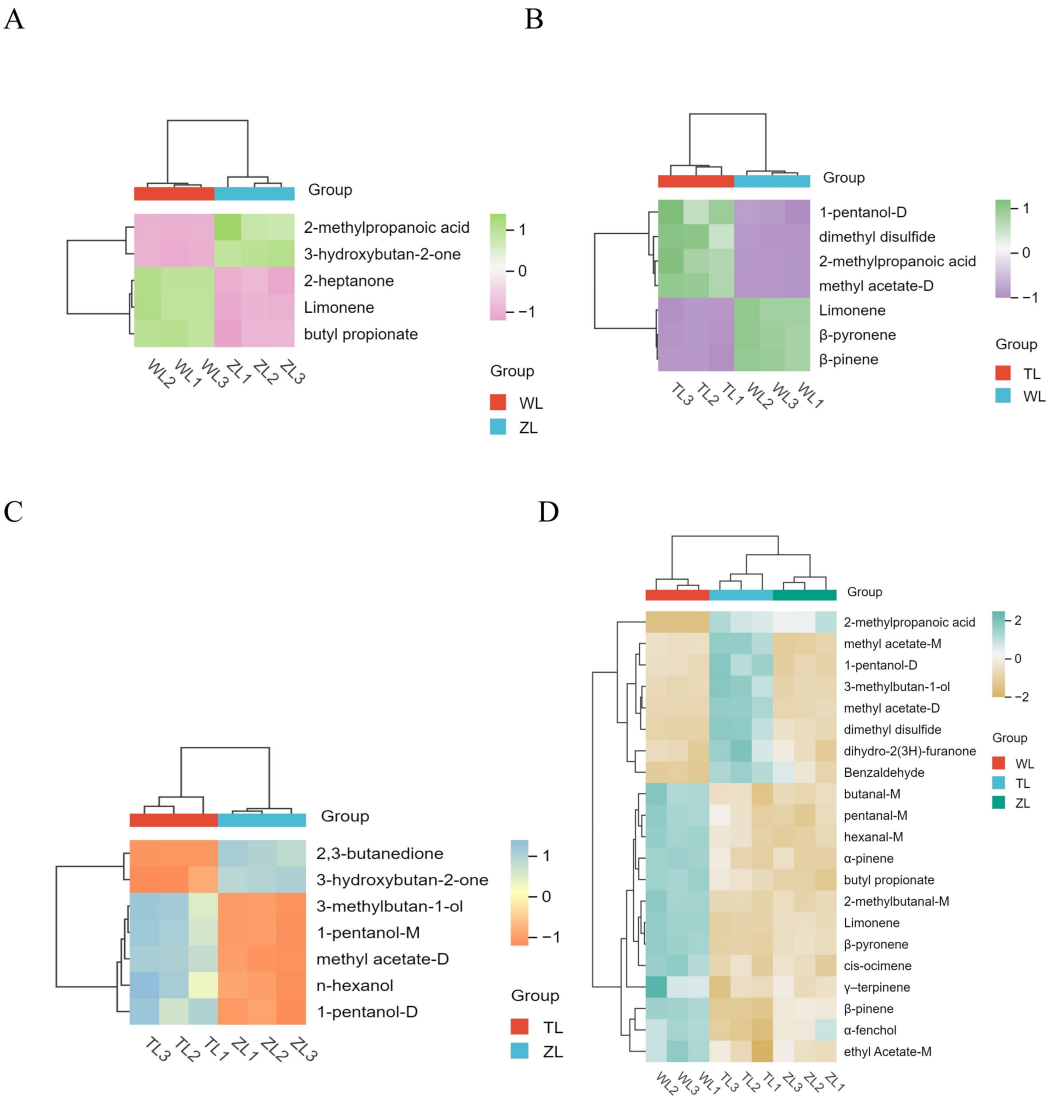
Each comparison group from volcano plot: **(A)** WL vs. ZL; **(B)** WL vs. TL; **(C)** ZL vs. TL. **(D)** Clustered heatmaps of a total of 21 VOCs with VIP > 1 (*p* < 0.05) across the three comparison groups. The lightly spicy chili powder was labeled Weila (WL), the medium spicy chili powder was labeled Zhongla (ZL), and the strongly spicy chili powder was labeled Tela (TL) in Chinese.

To clearly display the relative changes in differentially expressed compounds for each comparison group, as shown in Figures 5A–C, heatmaps were generated, where the depth of color indicates the relative abundance of each compound, facilitating an intuitive and systematic analysis and interpretation of the data on the differentially expressed flavor compounds. We also present clustered heatmaps of a total of 21 VOCs with VIP > 1 and *p* < 0.05 across the three comparison groups (Figure 5D), thereby visualizing the relative abundance differences of key flavor compounds across the group comparisons.

Supplementary Figures S1A–N box plots for a total of 14 VOCs, which are the compounds previously shown in the heatmaps of Figures 5A–C (under the selection criteria: VIP > 1, *p* < 0.05, FC > 2 or <0.5). The box plots illustrate the differences in peak volume and significance of these flavor compounds across the three chili powder groups (Supplementary Figures S1A–M). In the WL and TL groups, limonene, β-pinene, β-pyronene, methyl acetate-D, and butyl propionate presented very high levels of significance (*p* < 0.001) (Supplementary Figures S1A–E). Limonene provides a pleasant orange peel aroma (29). In the WL and ZL groups, 3-hydroxybutan-2-one was significantly different (*p* < 0.001) (Supplementary Figure S1F). Similarly, the level of 1-pentanol-M was significantly different (*p* < 0.001) between the ZL and TL groups (Supplementary Figure S1G). Studies have demonstrated that soil is among the factors influencing the spiciness of chili fruits (49), ripeness and variety (50), and storage methods (51). The chili powder used in this study was made by grinding chili peppers into a powdered form. This preparation process may accelerate the release of chili aromas, leading to the loss of volatile compounds. During the postpreparation storage phase, the increased surface area may accelerate the oxidation process, affecting its texture and flavor (52). These factors all significantly influence the flavor of chili powder.

### 3.7 Analysis of key flavor compounds in chili powders via the ROAV method

The odor detection threshold and odor-activity value (OAV) are crucial in assessing the contribution of volatile compounds to the aroma of food. Potent odorants typically have low odor detection thresholds, meaning that their odor can be detected at low concentrations. The OAV estimates odor strength by calculating the ratio between the concentration of a volatile compound and its odor detection threshold (53). The relative odor activity values (ROAVs) identify the compounds with the greatest contribution to the aroma by comparing the OAVs of all the substances and calculating their relative contribution ratios. According to the ROAV results (Supplementary Table S1), *(E, E)*-2,4-heptadienal, butanal, 3-methylbutanal, and 2,3-butanedione, with ROAVs > 1, are generally considered to make significant contributions to the overall odor characteristics of the sample (9). The chemical structures of these substances are displayed in Supplementary Figure S2. (*E, E*)-2,4-heptadienal, an aldehyde, has the greatest ROAV value and is characterized by its carbonyl group and high chemical reactivity. It possesses a distinctive grassy and fruity aroma and is considered a generally safe substance in the U. S. for use as a flavoring substance (54). Notably, this plant volatile compound has a molecular structure similar to that of traditional synthetic fungicides or preservatives. Recent studies have shown that *(E, E)*-2,4-heptadienal has a stronger inhibitory effect on *Aspergillus flavus* than does sorbaldehyde, thereby protecting stored peanut seeds from contamination by *A. flavus* (55). Supplementary Table S1 also shows that butanal, 2,3-butanedione, and 3-methylbutanal have slightly higher ROAVs in the medium spiciness level group (ZL). Each of these compounds results in distinct odor descriptions, a characteristic that may impart a certain uniqueness to the taste and flavor of medium spiciness chili powder.

Supplementary Figure S3 presents an odor wheel, with the inner circle representing the top 10 most prevalent sensory flavor characteristics found in all flavor compounds, including fatty, fruity, aldehydic, green, fermented, sweet, pungent, ethereal, herbal, and alcoholic flavors. The numbers in parentheses indicate the quantity of compounds associated with each flavor characteristic. The outer circle lists the compounds corresponding to these flavors. The interactions among these odors, such as masking, synergy, and inhibition, occur on multiple levels and collectively determine the complexity of the flavor of chili powder (56). The radar of the sensory aroma graph clearly revealed that fatty, fruity, aldehydic, and green flavors are more common, indicating that most compounds display these characteristic flavors (Supplementary Figure S4). Interestingly, the ROAV results (Supplementary Table S1) present significant discrepancies compared with the VIP rankings (Figure 4E). This is because the two methods focus on different aspects of analysis. The VIP score assesses the contribution of each variable to group differentiation and helps identify which compounds most strongly influence the classification of chili powders by spiciness level (57). In contrast, the relative odor activity value (ROAV) focuses on the sensory relevance of compounds by considering both their concentration and odor threshold. As a result, the ranking of compounds can differ substantially between these two methods. A compound may exhibit a high VIP score—indicating strong statistical relevance—while contributing minimally to aroma owing to a low ROAV, or vice versa. Therefore, integrating VIP and ROAV analyses offers a more comprehensive evaluation of aroma-active compounds by bridging statistical significance with sensory relevance, which is crucial for interpreting complex flavor profiles. Although the initial HS–GC–IMS fingerprints of VOCs in chili powder with different spiciness levels were obtained, providing a visual profile of the volatile components, further analysis via solid–phase microextraction combined with GC–O, SPME–GC–MS, olfactory techniques, and other flavor analysis techniques is needed. These findings provide more data for clarifying the flavor characteristics and establishing quality standards for chili powder.

## 4 Conclusion

In summary, the capsaicin contents and spiciness degrees of three commercial chili powders labeled with different spiciness levels (light, medium, and strong) were consistent. The E-nose effectively distinguished chili powders with different spiciness levels. Moreover, 48 volatile compounds, mainly aldehydes and ketones, were identified. PCA demonstrated that the cumulative contribution ratio of the first two components was 98.9%, implying that these principal components could effectively capture the major differences among the flavor compounds in these chili powders. Twenty-one marker volatiles were selected through OPLS-DA (VIP > 1, *p* < 0.05). Moreover, 14 differential flavor compounds between pairs of chili powder groups were screened according to fold change (FC > 2 or <0.5). *(E, E)*-2,4-heptadienal (grassy and fruity), butanal (pungent and green), 3-methylbutanal (ethereal and aldehydic), and 2,3-butanedione (sweet, creamy and pungent) are potential odor substances responsible for the variations in chili powders of different spiciness levels. This study lays the scientific groundwork for elucidating flavor differences among chili powders with varying degrees of spiciness. Further investigations focusing on detailed aroma profiling of these chili powders—using solid-phase microextraction combined with GC-O and SPME-GC–MS—should be presented in future reports.

## Data Availability

The original contributions presented in the study are included in the article/Supplementary material, further inquiries can be directed to the corresponding authors.

## Footnotes

^1^ https://www.metaboanalyst.ca/MetaboAnalyst/

^2^ https://cloud.metware.cn
