# Supplementary material for: Differentiation of volatile organic compounds in chili powders of different spiciness levels via E-nose, HS-GC–IMS, and chemometrics
Source: Front Nutr. 2025 Jul 28;12:1629925. doi: 10.3389/fnut.2025.1629925 (PMC12336983; doi:10.3389/fnut.2025.1629925)
Supplement: Supplementary file 1 [file Supplementary_file_1.docx]

Supplementary Material

**
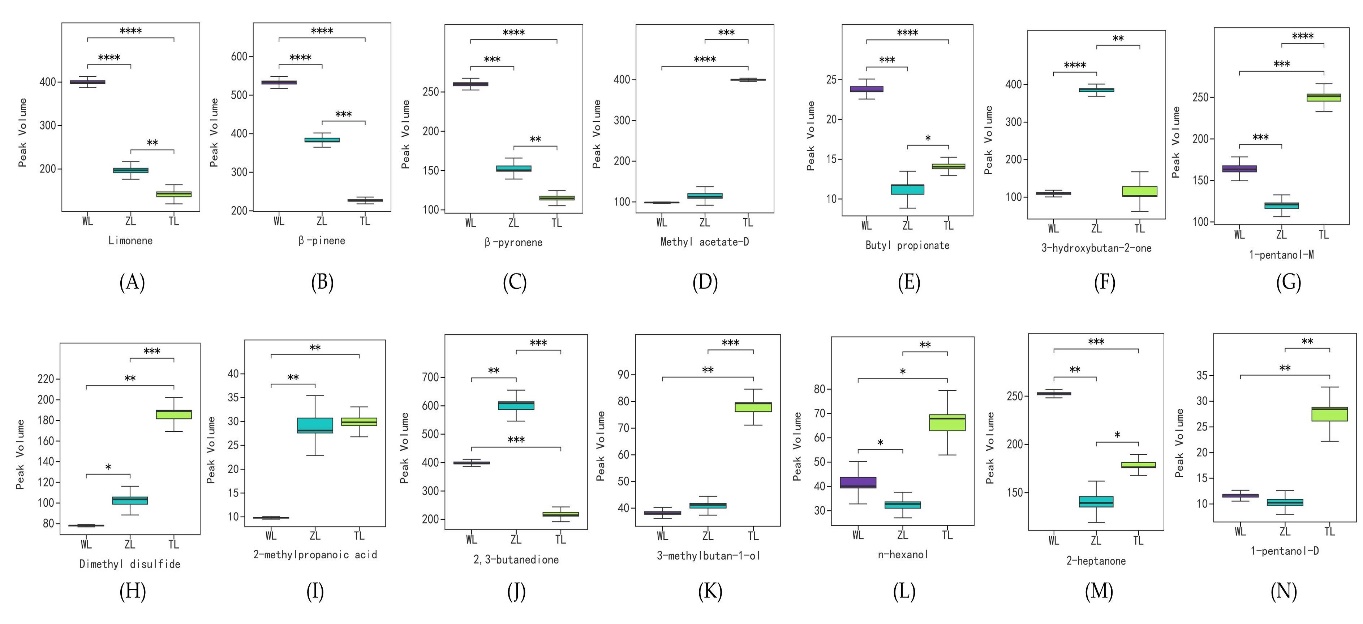
**

**Fig. S1** (**A-N**) Fourteen volatile organic compounds (under the selection criteria: VIP > 1, *p* < 0.05, FC > 2 or < 0.5) at different spiciness levels. The slightly spicy chili powder was labeled Weila (WL), the medium spicy chili powder was labeled Zhongla (ZL), and the strongly spicy chili powder was labeled Tela (TL) in Chinese.

**
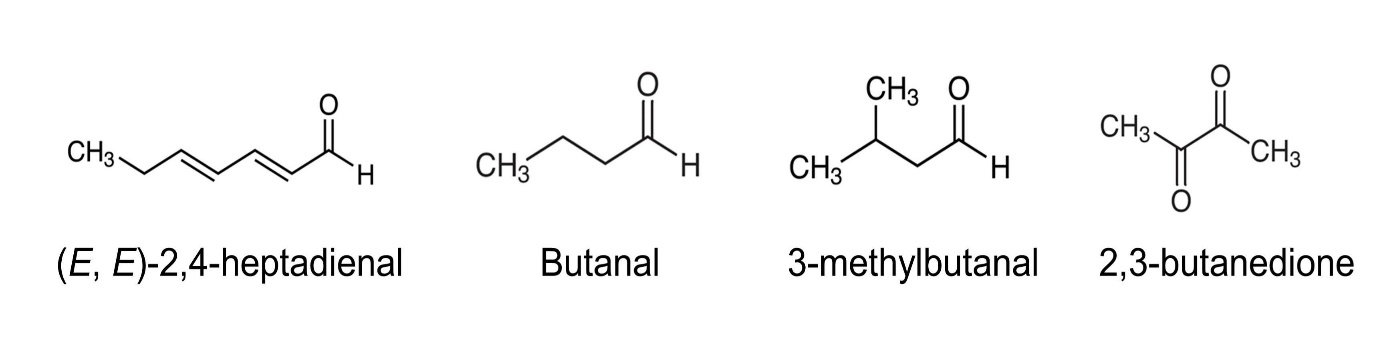
**

**Fig. S2** The chemical structures of volatile compounds with ROAVs > 1.

**
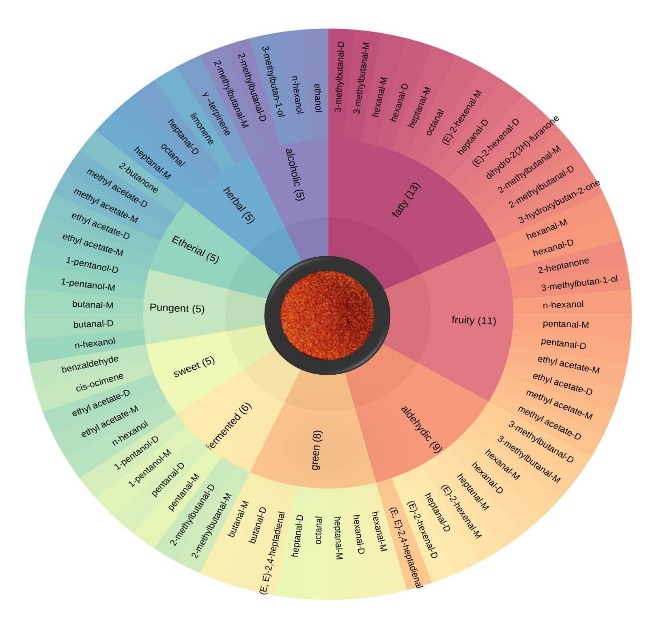
**

**Fig. S3** Odor wheel of the top ten most prevalent sensory flavor characteristics found in all flavor compounds.

**
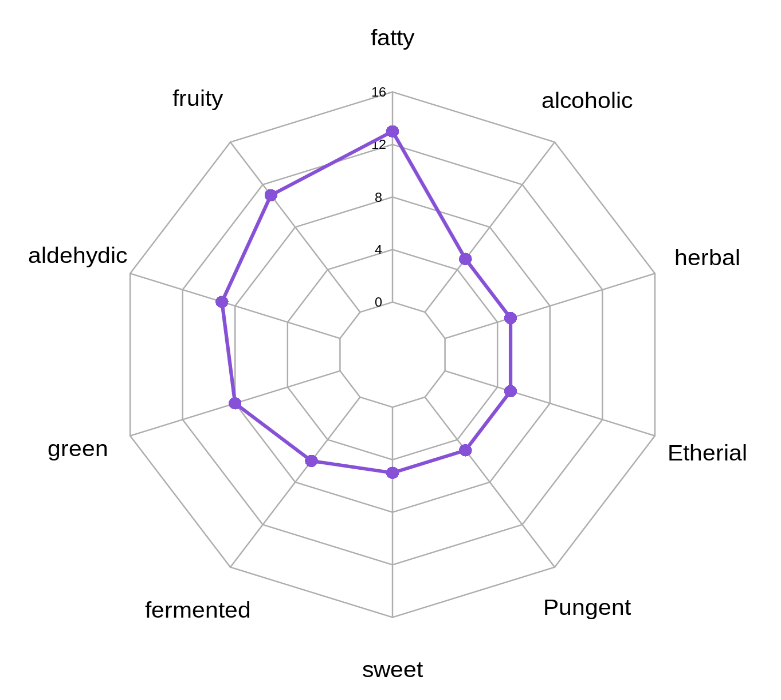
**

**Fig. S4** Radars of the sensory aroma graphs of the main sensory components at different levels of spiciness.

**Table S1.** Odor threshold in air and odor description of volatile organic compounds in chili powder with different spiciness levels

| No | compounds | relative odor activity value (ROAV) | | | Odor threshold (mg/m^3^) | odor description |
| --- | --- | --- | --- | --- | --- | --- |
|  |  | WL | ZL | TL |  |  |
| 1 | (*E*, *E*)-2,4-heptadienal | 100 | 100 | 100 | 0.0000006 | Fatty, green, oily, aldehydic, with a vegetative nuance |
| 2 | butanal-D | 3.529096006 | 6.095475417 | 3.530686353 | 0.00035 | Pungent, diffusive, cocoa, choking, musty, green, malty |
| 3 | 3-methylbutanal-D | 3.044276809 | 4.002995888 | 3.543113029 | 0.00035 | Ethereal, aldehydic, chocolate, peach, fatty |
| 4 | 2,3-butanedione | 2.192309945 | 3.279067858 | 1.382279894 | 0.00018 | Sweet, creamy, buttery, pungent, with a pungent caramellic nuance |
| 5 | butanal-M | 2.093402838 | 2.007796629 | 2.145953512 | 0.00035 | Pungent, diffusive, cocoa, choking, musty, green, malty |
| 6 | 3-methylbutanal-M | 1.270397735 | 1.063344865 | 1.323310598 | 0.00035 | Ethereal, aldehydic, chocolate, peach, fatty |
| 7 | 3-methylthiopropanal | 0.055650249 | 0.08337905 | 0.077076471 | 0.000063 | Musty, potato, tomato, earthy, vegetable, creamy |
| 8 | 2-heptanone | 0.010838799 | 0.006050199 | 0.008863451 | 0.023 | Cheese, fruity, ketonic, green banana, with a creamy nuance |
| 9 | dimethyl disulfide | 0.009162983 | 0.01198399 | 0.024978007 | 0.0084 | Sulfurous, vegetable, cabbage, onion |
| 10 | 3-methylbutan-1-ol | 0.006191685 | 0.006603532 | 0.01442105 | 0.0061 | Fusel, alcoholic, pungent, ethereal, cognac, fruity, banana, molasses |
| 11 | hexanal-M | 0.005517195 | 0.004350784 | 0.005014424 | 0.23 | Fresh, green, fatty, aldehydic, grass, leafy, fruity, sweaty |
| 12 | hexanal-D | 0.004110528 | 0.002956718 | 0.003732168 | 0.23 | Fresh, green, fatty, aldehydic, grass, leafy, fruity, sweaty |
| 13 | *β*-pinene | 0.002919698 | 0.00211146 | 0.001431642 | 0.18 | Herbal, pine |
| 14 | cis-ocimene | 0.002707841 | 0.001908547 | 0.002012602 | 0.01 | Warm, floral, herb, flower, sweet |
| 15 | (*E*)-ocimene | 0.002702796 | 0.002422116 | 0.003280626 | 0.0187 | Floral |
| 16 | acetone | 0.002115818 | 0.002289801 | 0.002379923 | 2 | Solvent, ethereal, apple, pear |
| 17 | limonene | 0.001882886 | 0.0009245 | 0.00076441 | 0.21 | Citrus, herbal, woody |
| 18 | 2-methylpropanoic acid | 0.00178881 | 0.005388995 | 0.006305605 | 0.0054 | Acidic, sour, cheese, dairy, buttery, rancid |
| 19 | benzaldehyde | 0.001471649 | 0.002135799 | 0.003098728 | 0.085 | Fruity, strong, sharp, sweet, bitter, almond, cherry |
| 20 | n-hexanol | 0.001221252 | 0.000933861 | 0.002194623 | 0.034 | Pungent, ethereal, fusel oil, fruity, alcoholic, sweet, with a green top note |
| 21 | ethanol | 0.000763981 | 0.000516262 | 0.000711288 | 0.62 | Strong, alcoholic, ethereal, medical |
| 22 | pentanal-M | 0.000577544 | 0.000481761 | 0.000553611 | 0.85 | Diffusive, fermented, bready, fruity, with berry nuances |
| 23 | *α*-pinene | 0.000482771 | 0.000321008 | 0.000360924 | 0.1 | Herbal, woody |
| 24 | 1-pentanol-M | 0.000450374 | 0.000326602 | 0.00078646 | 0.36 | Pungent, fermented, bready, yeasty, fusel, winey, solvent-like |
| 25 | pentanal-D | 0.000434035 | 0.000419597 | 0.000495854 | 0.85 | Diffusive, fermented, bready, fruity, with berry nuances |
| 26 | heptanal-M | 0.000369261 | 0.000331043 | 0.000402589 | 0.26 | Fresh, aldehydic, fatty, green, herbal, cognac, ozone |
| 27 | octanal | 0.000291674 | 0.000314183 | 0.000351725 | 0.17 | Aldehydic, waxy, citrus, orange peel, green, herbal, fresh, fatty |
| 28 | ethyl acetate-M | 0.000243831 | 0.000242434 | 0.000249665 | 0.88 | Etherial, fruity, sweet, grape, rum-like |
| 29 | butyl propionate | 0.000124327 | 5.71704E-05 | 8.43636E-05 | 0.19 | Sweet, estry, banana, ripe, tufti-frutti, bright, lifting, cherry, rum-like |
| 30 | ethyl acetate-D | 0.00010797 | 0.000111742 | 0.000117355 | 0.88 | Etherial, fruity, sweet, grape, rum-like |
| 31 | 2-butanone | 6.85439E-05 | 0.000101196 | 7.34643E-05 | 1.3 | Etherial, diffusive, and slightly fruity, with a camphorous nuance |
| 32 | (*E*)-2-hexenal-M | 5.40061E-05 | 4.05207E-05 | 7.43972E-05 | 0.79 | Green, banana, aldehydic, fatty, cheesy |
| 33 | heptanal-D | 4.18197E-05 | 3.86844E-05 | 4.36157E-05 | 0.26 | Fresh, aldehydic, fatty, green, herbal, wine-lee, ozone |
| 34 | methyl acetate-M | 3.93611E-05 | 3.69924E-05 | 7.73167E-05 | 5.1 | Etherial, solvent-like, estry, fruity, winey, cognac, rum |
| 35 | 1-pentanol-D | 3.16082E-05 | 2.82675E-05 | 8.56539E-05 | 0.36 | Pungent, fermented, bready, yeasty, fusel, winey, solvent-like |
| 36 | furfural | 2.06435E-05 | 2.27177E-05 | 2.10552E-05 | 2.8 | Sweet, woody, almond, fragrant, baked, bread |
| 37 | methyl acetate-D | 1.89841E-05 | 2.22978E-05 | 0.000089079 | 5.1 | Etherial, solvent-like, estry, fruity, winey, cognac, rum |
| 38 | (*E*)-hept-2-enal-M | 1.78872E-05 | 1.72976E-05 | 2.17265E-05 | 2.4 | - |
| 39 | (*E*)-2-hexenal-D | 9.26266E-06 | 8.39778E-06 | 1.39883E-05 | 0.79 | Green, banana, aldehydic, fatty, cheesy |
| 40 | (*E*)-hept-2-enal-D | 6.63334E-06 | 6.89061E-06 | 8.99894E-06 | 2.4 | - |
| 41 | *γ* –terpinene | 5.49406E-07 | 4.60023E-07 | 4.50331E-07 | 55 | Oily, woody, terpene, lemon, lime, tropical, herbal |
| 42 | *β*-pyronene | - | - | - | - | - |
| 43 | *α*-fenchol | - | - | - | - | Earthy, spicy, notes |
| 44 | dihydro-2(3H)-furanone | - | - | - | - | Creamy, oily, fatty, caramel |
| 45 | (*E*)-2-hexenol | - | - | - | - | - |
| 46 | 2-methylbutanal-M | - | - | - | - | Musty, cocoa, phenolic, coffee, nutty, malty, fermented, fatty, alcoholic |
| 47 | 2-methylbutanal-D | - | - | - | - | Musty, cocoa, phenolic, coffee, nutty, malty, fermented, fatty, alcoholic |
| 48 | 3-hydroxybutan-2-one | - | - | - | - | Sweet, buttery, creamy, dairy, milky, fatty |

The threshold data for the compounds primarily come from this book: *Compilations of Odour Threshold Values in Air, Water & Other Media AND Compilations of Flavour Threshold Values in Water & Other Media (2011 Editions)* by L.J. van Gemert. Odor descriptions were sourced from online websites www.femaflavor.org and www.thegoodscentscompany.com. Some threshold values and odor descriptions remain unavailable.
